# Corrigendum

**DOI:** 10.1002/ccr3.7047

**Published:** 2023-04-11

**Authors:** Alexis Rafael Narvaez‐Rojas

In the article entitled “Indocyanine green fluorescence: A surgeon's tool for the surgical approach of gallstone ileus” that was previously published in Volume 10 Issue 5 of Clinical Case Reports, the author's list was incorrect in the published version.

The correct author list should be as follows: Ossian Fuentes, Nicolas Arredondo, Ivan David Lozada‐Martinez, Daniela Guardo‐Carmona, Alexis Rafael Narvaez‐Rojas, Luis Felipe Cabrera‐Vargas.

We apologize for the error.
